# Direct Transmembrane Interaction between Actin and the Pore-Competent, Cholesterol-Dependent Cytolysin Pneumolysin

**DOI:** 10.1016/j.jmb.2012.11.034

**Published:** 2013-02-08

**Authors:** Sabrina Hupp, Christina Förtsch, Carolin Wippel, Jiangtao Ma, Timothy J. Mitchell, Asparouh I. Iliev

**Affiliations:** 1 DFG Membrane/Cytoskeleton Interaction Group, Institute of Pharmacology and Toxicology & Rudolf Virchow Centre for Experimental Biomedical Science, University of Würzburg, Versbacherstr. 9, 97078 Würzburg, Germany; 2 Institute of Infection, Immunity and Inflammation, College of Medical, Veterinary and Life Sciences, University of Glasgow, 120 University Place, Glasgow G12 8TA, Scotland, United Kingdom; 3 Chair of Microbial Infection and Immunity, School of Immunity and Infection, College of Medical and Dental Sciences, University of Birmingham, Edgbaston, Birmingham B15 2TT, United Kingdom

**Keywords:** PLY, pneumolysin, Δ6-PLY, non-pore-forming mutant of pneumolysin, CDC, cholesterol-dependent cytolysin, GUV, giant unilamellar vesicle, FRET, Förster resonance energy transfer, FRAP, fluorescence recovery after photobleaching, APB, actin polymerisation buffer, GFP, green fluorescent protein, PBS, phosphate-buffered saline, RT, room temperature, actin, cholesterol-dependent cytolysin, membrane, pneumolysin, pore-forming toxin

## Abstract

The eukaryotic actin cytoskeleton is an evolutionarily well-established pathogen target, as a large number of bacterial factors disturb its dynamics to alter the function of the host cells. These pathogenic factors modulate or mimic actin effector proteins or they modify actin directly, leading to an imbalance of the precisely regulated actin turnover. Here, we show that the pore-forming, cholesterol-dependent cytolysin pneumolysin (PLY), a major neurotoxin of *Streptococcus pneumoniae*, has the capacity to bind actin directly and to enhance actin polymerisation *in vitro*. In cells, the toxin co-localised with F-actin shortly after exposure, and this direct interaction was verified by Förster resonance energy transfer. PLY was capable of exerting its effect on actin through the lipid bilayer of giant unilamellar vesicles, but only when its pore competence was preserved. The dissociation constant of G-actin binding to PLY in a biochemical environment was 170–190 nM, which is indicative of a high-affinity interaction, comparable to the affinity of other intracellular actin-binding factors. Our results demonstrate the first example of a direct interaction of a pore-forming toxin with cytoskeletal components, suggesting that the cross talk between pore-forming cytolysins and cells is more complex than previously thought.

## Graphical Abstract

**Figure:**
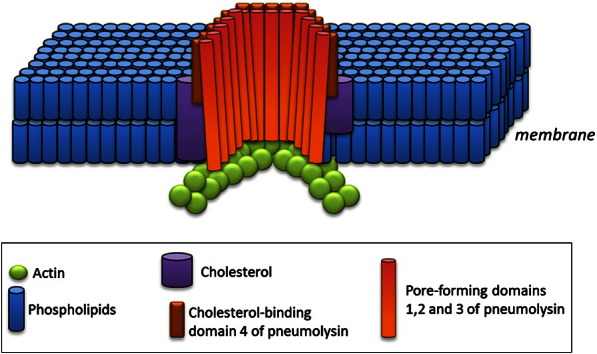

**Highlights:**

► The cholesterol-dependent cytolysin PLY interacts with actin with high affinity. ► PLY stabilises actin. ► Pore formation is critically important for the interaction with actin. ► The interaction can occur through the membrane bilayer. ► The pore formation by PLY allows toxin access to intracellular targets.

## Introduction

**Fig. 1:**
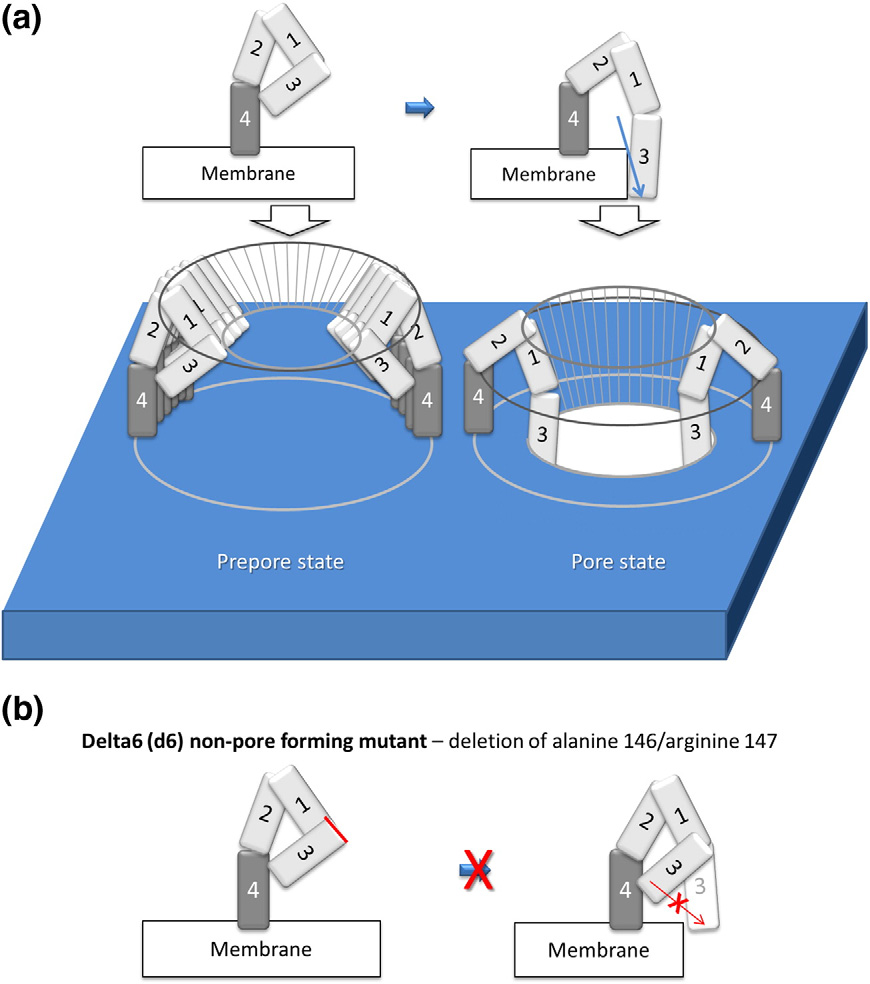
(a) Schematic representation of the pre-pore and pore formation by PLY and of the orientation of individual domains (1, 2, 3 and 4) in this process (according to Tilley *et al.*^2^). (b) Schematic representation of the non-pore-forming Δ6-PLY mutant.

Bacteria produce effector molecules, such as protein toxins, to modulate host cell function and host defence, thereby increasing bacterial pathogenic potential. Many bacterial effector molecules are pore-forming proteins, which build holes in membranes, thereby damaging cells. The cholesterol-dependent cytolysin (CDC) pneumolysin (PLY), a major virulence factor of *Streptococcus pneumoniae* (pneumococcus), produces lytic membrane pores at high concentrations and smaller, presumably non-lytic, pores at lower concentrations.^1^ The formation of pores involves a cholesterol-binding step, in which the toxin molecules oligomerise into a barrel structure of 30–50 monomers (Fig. 1a). The cholesterol-binding step is followed by a penetration step, in which components of the oligomerised toxin molecules unfold and penetrate the membrane to form the pore.^2^ PLY comprises four domains. Domain 4 recognises and binds to cholesterol in the membrane, while domains 1, 2 and 3 remain external to the membrane in an initial pre-pore ring structure (schematically depicted in Fig. 1a). Upon oligomerisation in a pre-pore ring, a complex conformational change occurs.^2,3^ Domain 2 bends towards the membrane, allowing the subsequent partial refolding of domain 3. In this process, the helical regions of domain 3, hidden inside the molecule, are refolded into two β-hairpins (each containing two parallel β-strands), which penetrate the membrane and build the internal β-barrel of the pore^2^ (schematically depicted in Fig. 1a). For experimental and immunisation purposes, a non-pore-forming mutant (Δ6-PLY)^4^ and the fragments D4-PLY (containing domain 4) and D123-PLY (containing domains 1, 2 and 3) have been created. Δ6-PLY has been shown to preserve membrane-binding activity,^5^ but it lacks the amino acids alanine 146 and arginine 147 at the transition interface from domain 1 to domain 3 (Fig. 1b), rendering the toxin non-lytic due to a compromised refolding and penetration capacity of domain 3 (Fig. 1b).

In the pore conformation, PLY penetrates the membrane and can either lyse the cells immediately or produce ion fluxes and cell shape remodelling.^1,5,6^ The exact factors determining which cells survive and which cells are lysed remain elusive. It is known that cells possess membrane repair mechanisms, such as the internalisation of damaged membrane fragments and pores and replacement with new intact membranes, which most likely contribute to the resistance against the effect of lytic PLY molecules.^7,8^

Our earlier finding of actin cytoskeletal modulation *via* the activation of small GTPases by PLY identified a surprising function of a CDC,^9^ but one that is typical of many other bacterial effectors. PLY is the only known pore-forming toxin that alters the actin cytoskeleton *via* small GTPases in intact cells. The activation of the small GTPases RhoA and Rac1 by PLY is a complex phenomenon that involves protein kinase C and membrane depolarisation and that requires intact pore-forming capacity.^9^ In cell culture conditions, shape changes and actin remodelling are observed even when using low concentrations of PLY that lyse less than 5% of all cells (we have defined these concentrations as sub-lytic). The Δ6 non-pore-forming toxin mutants do not affect cell shape or the actin cytoskeleton, suggesting a role of pore formation capacity in the shape alterations in surviving cells.^5^

The eukaryotic actin cytoskeleton is an evolutionarily well-established pathogen target, as a large number of bacterial effector proteins alter it. Some toxins, such as the binary and large clostridial glucosylating toxins, the Tc toxins of *Photorhabdus luminescens* and the actin cross-linking toxins, affect actin directly.^10^ Other toxins change the function of actin-regulating small GTPases by modifying them covalently or by mimicking small GTPase-regulating factors.^11^ Some toxins have adapted to interact with actin-binding proteins, such as the Arp2/3 complex and its regulators.^12^

The aim of the current work was to study the possibility of a direct interaction between actin and PLY in the first minutes after toxin exposure.

## Results

### PLY and actin co-localise transiently at the plasma membrane shortly after toxin challenge

**Supplementary Fig. S1:**
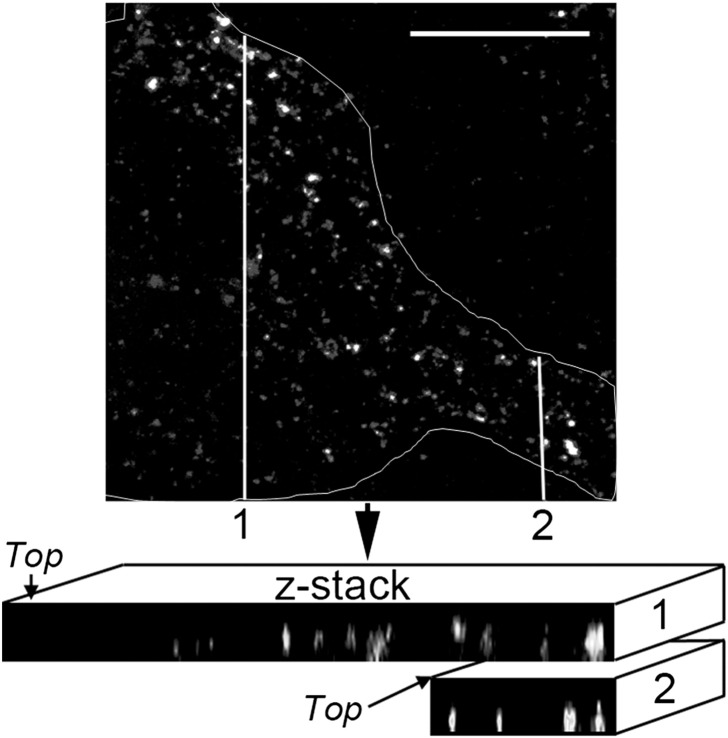
Confocal microscopy of mouse astrocytes that were challenged with 0.5 μg/ml WT-PLY-GFP and that demonstrated vesicle-like intracellular localisation at 240 s, which was confirmed by a z-stack reconstruction. The white contour outlines the borders of the astrocyte. Scale bars represent 10 μm.

**Fig. 2:**
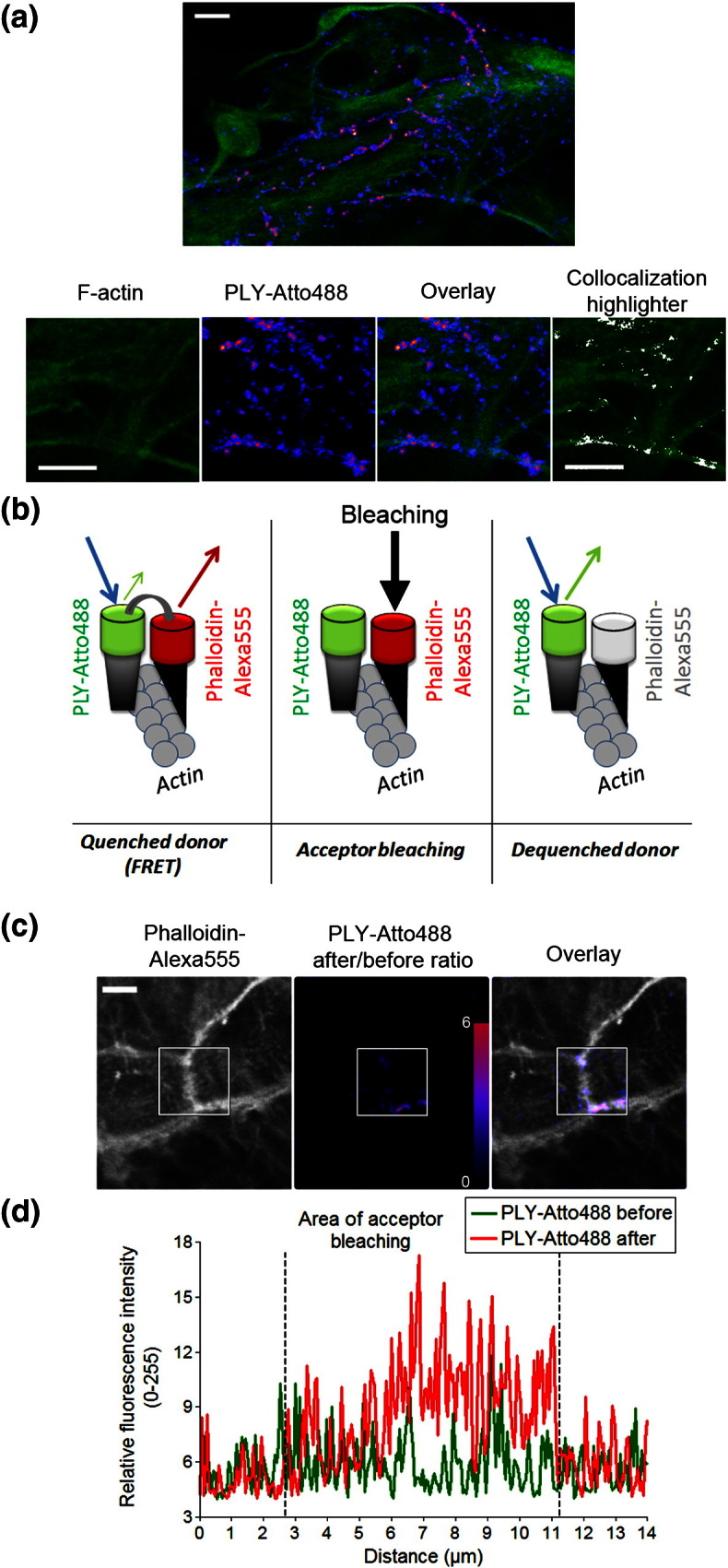
Actin and PLY co-localisation and interaction in astrocytes. (a) Co-localisation between F-actin [stained with phalloidin (green)] and PLY [Atto488-tagged, 0.2 μg/ml (blue–magenta)] in astrocytes 90 s after toxin exposure (a single confocal plane along the surface of the cell). The co-localisation highlighter plugin from ImageJ is applied with standard settings (ratio, 50%; channel threshold, 50). The scale bar represents 10 μm. (b) Principle of FRET measurements *via* acceptor photobleaching. In the case of FRET (at the molecular interaction distance between the donor and the acceptor), the donor fluorescence is quenched by the acceptor. Upon bleaching of the acceptor, the donor is de-quenched, and its fluorescence increases. PLY is labelled with Atto488 (PLY-Atto488; donor), and F-actin is labelled with phalloidin-Alexa555 (acceptor). (c) Phalloidin staining of cortical F-actin in an astrocyte monolayer, showing the border region fragments of four cells tightly attached to each other. The ratio of donor fluorescence before/after bleaching of the acceptor demonstrates the presence of strong FRET along the phalloidin-positive subcortical F-actin structures 120 s after the PLY-Atto488 challenge. The scale bar represents 10 μm. (d) Representative profile scan along the cortical actin structures shown in (c) demonstrating the changes in donor fluorescence after bleaching.

We used primary mouse astrocytes as a model cell system because astrocytes represent the natural environment of pneumococcal meningitis, where PLY plays a key role.^13^ Similar to our earlier report of small GTPase activation by PLY in human neuroblastoma cells,^9^ PLY also activated RhoA and Rac in astrocytes by 4 min after the toxin challenge (not shown). Therefore, we followed the distribution of fluorescent Atto488-labelled PLY and actin in the first 4 min (Fig. 2a). Ninety seconds after toxin application, more than the half of all PLY-Atto488 puncta co-localised with the actin bundles positioned directly beneath the plasma membrane of the cells (Fig. 2a, single confocal plane). Confocal z-stack reconstruction revealed that the toxin puncta were positioned close to the surface of the cells. To study whether this co-localisation represented a direct interaction, we utilised a Förster resonance energy transfer (FRET) approach, namely, acceptor photobleaching (Fig. 2b), which was able to show the interaction of both molecules. F-actin was labelled with phalloidin-Alexa555 (as the acceptor) in fixed cells exposed to PLY-Atto488 (as the donor) before fixation (Fig. 2b). In the first 60 s of PLY-Atto488 exposure, no FRET was observed after acceptor photobleaching (not shown). In the 90–240 s after toxin exposure, FRET was observed in three of five cells following acceptor photobleaching, specifically along the cortical actin structures (Fig. 2c and d), which was indicative of the proximity between PLY and phalloidin-labelled actin being in the range of the molecular interaction distance (< 10 nm). At later time points (> 240 s), FRET was no longer detected (not shown); however, PLY was observed internalised in vesicular structures (Supplementary Fig. S1), and no substantial co-localisation with actin was observed. Thus, a direct interaction between PLY and actin was observed within the first 4 min after treatment with the toxin, and this interaction was of a transient (i.e., the interaction only lasted several minutes) nature.

### PLY binds actin with high affinity

**Fig. 3:**
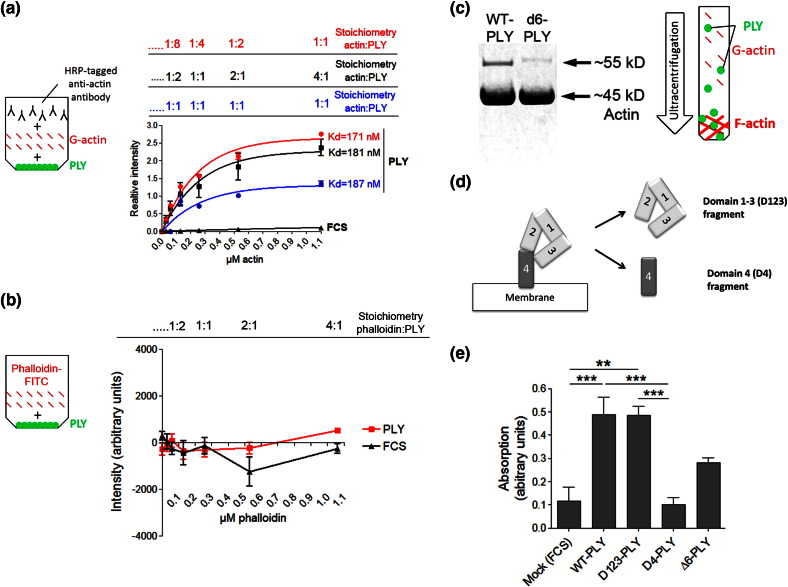
Biochemical characterisation of PLY binding to actin. (a) ELISA-based measurement of the G-actin affinity to PLY (measured by the dissociation constant *K*_d_) in various stoichiometric ratios, which produce identical results. *n* = 3–5 measurements per ratio. (b) Lack of phalloidin binding to PLY, as measured by the fluorescence of phalloidin-Alexa488. *n* = 4 measurements (for a rough orientation of the intensity scale, the intensity of the initially applied phalloidin-Alexa488 at 260 μM and a volume of 50 μl exceeded 60,000 A.U.). (c) A representative experiment (performed in triplicate with identical results) demonstrating the co-sedimentation of F-actin with wild-type PLY (WT-PLY) and the reduced co-sedimentation of the PLY Δ6-mutant. (d) Schematic presentation of the fragments D123 and D4, which were utilised in the binding ELISA assay in (e), according to the toxin domains they comprise. (e) Analysis of G-actin binding to different forms and fragments of PLY. The actin concentration is 2.2 μM, corresponding to the plateau of the affinity curve in (a). G-acting binds strongest to WT-PLY and to the domain 1–3 fragment of PLY (D123-PLY) and not to the domain 4 fragment (D4-PLY). The Δ6-PLY mutant shows intermediate binding. ^⁎⁎^*p* < 0.01, *^⁎⁎⁎^p* < 0.001, *n* = 6. All the values represent the mean ± SEM.

Biochemical quantification of the affinity of the interaction between PLY and actin allows for a comparison with other known actin-binding factors. A suitable marker for the affinity of this interaction is the dissociation constant *K*_d_. Lower *K*_d_ values correspond to higher-affinity interactions. We developed an ELISA-based assay in which PLY-coated surfaces were exposed to varying concentrations of G-actin or fluorescently labelled phalloidin (Fig. 3a and b). G-actin bound PLY with a high affinity, demonstrating a *K*_d_ of 170–190 nM (Fig. 3a); phalloidin did not bind PLY (Fig. 3b). These results verified that the FRET experiments detected an interaction between PLY and actin and not with phalloidin. To verify that the *K*_d_ for the affinity of actin binding to PLY was not falsely derived due to stoichiometric saturation (i.e., when no more binding positions on PLY molecules were available due to the binding of excessive amounts of actin), we repeated the experiment at several different stoichiometric ratios (changing the amount of coated PLY) and obtained identical results (Fig. 3a; for the exact protocol details, see Materials and Methods). PLY also bound preformed F-actin filaments and co-sedimented with them after ultracentrifugation (Fig. 3c). The co-sedimentation of the non-pore-forming toxin mutant Δ6-PLY with actin filaments was substantially reduced compared to the co-sedimentation observed with wild-type PLY (Figs. 3c and 1b and Introduction).^4^

### The domains of PLY critical for pore formation are also essential for the interaction of PLY with actin

We analysed individual fragments of PLY to determine which domains are mechanistically necessary for actin binding. We expanded the ELISA interaction assay to include the Δ6-PLY mutant and PLY fragments. We used two fragments of PLY: one containing domain 4 only (domain 4 binds cholesterol in the membrane but does not penetrate it; see Figs. 1a and 3d) and another fragment comprising domains 1, 2 and 3, which are critically important for the ability of the toxin to penetrate the membrane (see Figs. 1 and 3d). The strongest binding was demonstrated by the fragment containing domains 1, 2 and 3, compared to the domain 4 fragment (Fig. 3e). The Δ6-PLY mutant bound actin more weakly than wild-type PLY but bound actin stronger than the mock control (Fig. 3e). These experiments indicated that the toxin domains and amino acids that are involved in and are critical for PLY pore formation are also important for interaction of the PLY with actin.

### PLY enhances actin polymerisation

**Fig. 4:**
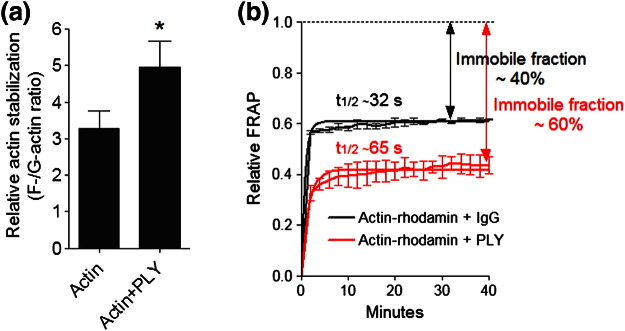
Increased actin polymerisation in the presence of PLY. (a) Increased actin stabilisation (increased F-actin in the protein pellet *versus* G-actin in the solution after sedimentation of F-actin by ultracentrifugation at 150,000***g*** for 90 min) in the presence of 600 nM PLY for 1 h at 24 °C. The amount of actin was measured by anti-actin Western blot analysis. ^⁎^*p* > 0.05, *n* = 5 experiments. (b) Increased actin polymerisation caused by PLY, but not by IgG (control protein), in a biochemical system, as determined by FRAP analysis of actin-rhodamine 60 min after exposure to the APB (which provides Mg^2 +^ and ATP). After bleaching of the actin-rhodamine in solution (for 5 s at maximum 561 nm laser power), the change in fluorescence due to diffusion was followed in the bleached area. The longer half-time of fluorescence recovery indicates larger and more slowly diffusing fragments. The larger immobile fraction indicates a larger, completely immobile and presumably highly polymerised fraction. *n* = 4 experiments. All the values represent the mean ± SEM.

Next, we addressed whether PLY binding to actin can also alter actin dynamics, namely, actin polymerisation and depolymerisation. We analysed the F-actin/G-actin ratio following the polymerisation of actin in actin polymerisation buffer (APB) in the absence and presence of PLY. PLY enhanced the polymerisation of the monomeric G-actin into F-actin (Fig. 4a). A similar effect was observed when fluorescence recovery after photobleaching (FRAP) was performed on rhodamine-labelled actin in APB. The FRAP approach is a powerful tool for studying the level of polymerisation of fluorescent molecules, as the molecular diffusion (measured by the half-time of fluorescence recovery) decreases as the size of the oligomers increases. The formation of large non-diffusing fluorescent polymers is detected as an immobile fraction (i.e., the portion that does not diffuse and does not recover fluorescence after bleaching^14^). The longer half-time of recovery of fluorescent actin in the presence of PLY and the increased immobile fraction indicated stronger actin oligomerisation and polymerisation, respectively, compared to actin in the presence of IgG (as a control protein) (Fig. 4b). This result confirmed that PLY not only binds actin but can also interfere with its polymerisation status.

### PLY interacts with actin through lipid bilayers

**Supplementary Fig. S2:**
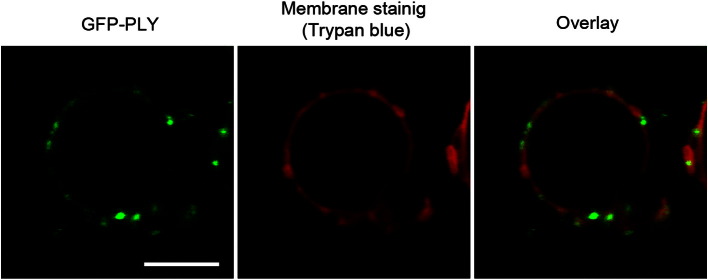
GFP-PLY orientation in the GUV membrane. Trypan blue (a GFP quencher) applied outside the GUVs stained the lipid bilayer red but did not quench GFP fluorescence. This result indicates the intravesicular localisation of the N-terminus of the toxin, where GFP is localised (as represented schematically in Fig. 5a). The scale bar represents 10 μm.

**Fig. 5:**
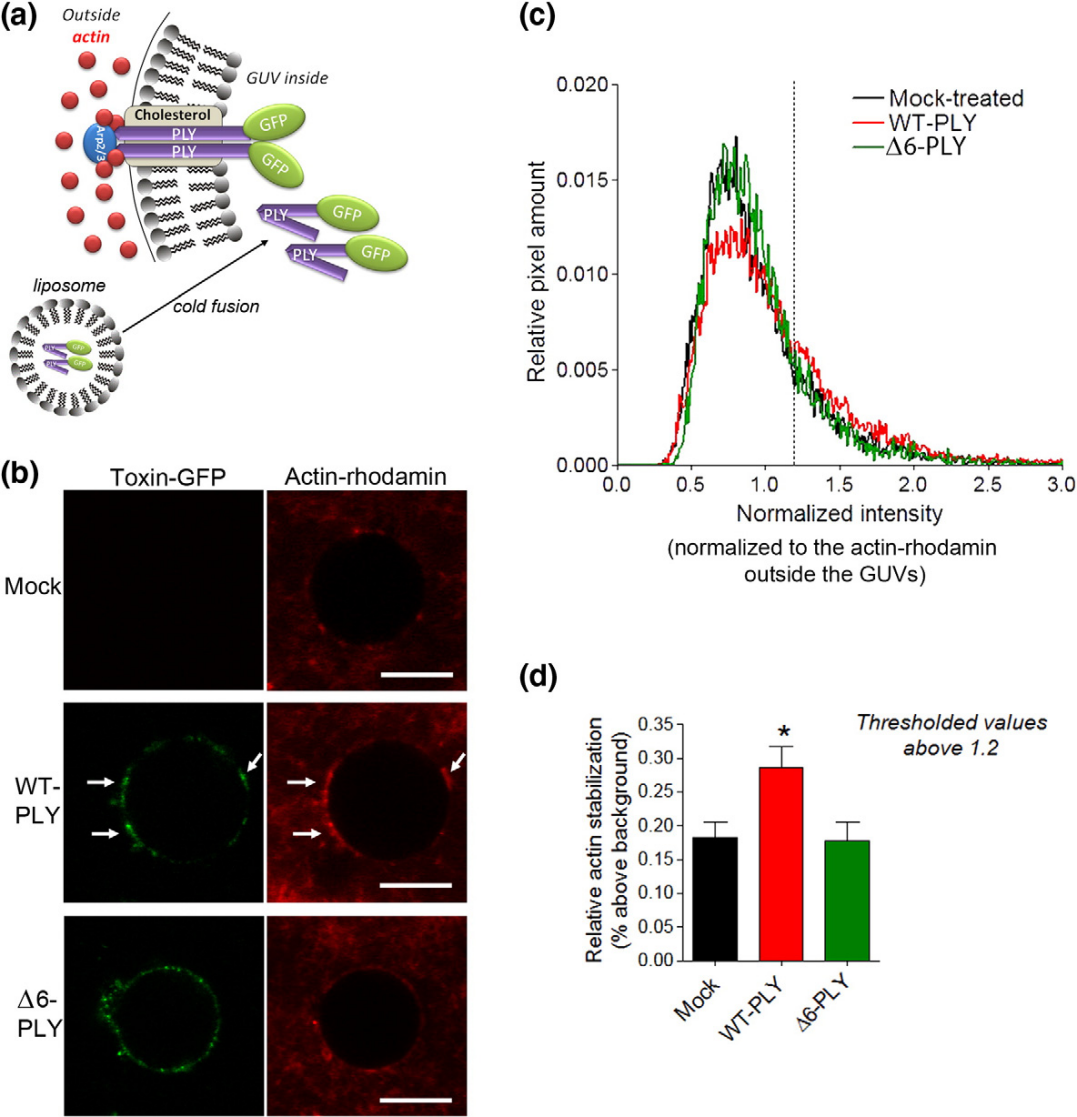
Transmembrane actin clustering by PLY in GUVs. (a) Schematic representation of the GUV experimental approach for the fusion of liposomes (for a complete description, see Materials and Methods). The toxin and the actin molecules are positioned on opposite sides of the lipid bilayer. (b) Aggregation of actin-rhodamine on the surface of the GUVs in close proximity to the PLY-GFP clusters is observed with wild-type PLY (WT-PLY; arrows), but not in the mock sample or with the non-pore-forming Δ6-PLY GUVs. The scale bar represents 10 μm. (c) Cumulative histogram of the pixel intensity distribution along the GUV surface normalised to the average actin-rhodamine staining intensity outside the GUV (measured at a distance of one diameter from the surface of the GUV). The histogram represents the cumulative data from 15 to 20 GUVs in one representative experiment (the mean only). The experiment was repeated three times with identical results. (d) Increased actin aggregation (as measured by the increased rhodamine staining intensity above the actin background) in the WT-PLY GUVs. ^⁎^*p* < 0.05; the values represent the mean ± SEM of 15–20 GUVs within one representative experiment.

Affinity measurement experiments and the analysis of actin polymerisation were performed under conditions in which no lipid bilayer separated PLY from its interacting partners. However, such a membrane barrier does exist in cells. To test whether PLY and actin can interact directly through a membrane bilayer devoid of additional traffic/repair properties, we utilised a giant unilamellar vesicle (GUV) approach.^15^ In this system, giant (5–30 μm in diameter) membrane bilayer vesicles were prepared by electroformation.^16^ The components of the system were limited to phospholipids and cholesterol in the membrane, rhodamine-labelled actin and the Arp2/3 protein complex outside of the GUVs and PLY-green fluorescent protein (GFP) within the GUVs. The loading of PLY-GFP inside the GUVs was achieved using a liposome carrier (see Materials and Methods) *via* 4 °C fusion^16^ (Fig. 5a). With this approach, we reproduced the orientation of PLY and actin on both sides of the bilayer. No free PLY or PLY that bound the GUVs on the external surface was present in the system (see Materials and Methods and Supplementary Fig. S2). Actin was incubated in APB to provide the ATP and Mg^2 +^ necessary for polymerisation, which, together with Arp2/3, allowed for slow but detectable spontaneous polymerisation. This polymerisation was visible as small actin clusters on the surface of the GUVs after 30–60 min. Thus, this system allows for the analysis of both actin stabilisation and actin destabilisation, depending on how and if the PLY-GFP has an effect. We observed that wild-type PLY-GFP accelerated the aggregation and polymerisation of actin-rhodamine on the GUV surface (Fig. 5b–d), as PLY-GFP puncta co-localised with the largest actin-rhodamine clusters (Fig. 5b, arrows). The non-pore-forming Δ6-GFP mutant, which also partially clustered in the membrane (fluorescent puncta in Fig. 5b), did not produce the same effects on actin-rhodamine as the wild-type PLY-GFP did. These GUV experiments confirmed the ability of PLY to target actin through the lipid bilayer, but only when the toxin can form pores.

## Discussion

The aim of the current work was to obtain mechanistic insights into the earliest effects of PLY on cellular actin. The results revealed selective and high-affinity (*K*_d_ of 170–190 nM) binding of PLY to actin and the ability of PLY to enhance actin polymerisation *in vitro*. The effects of PLY were dependent on its pore formation capacity, suggesting that the active actin-interacting partner is the toxin in a pore state. The interaction was transient, occurring shortly after PLY binding to the membrane and ceasing with the internalisation of the toxin. The actin interaction described here represents the first evidence for a direct interaction between a pore-forming toxin and actin. These results complement our previous findings of small GTPase-dependent cellular modifications by PLY that occur after longer (> 4 min)^9^ exposure to the toxin. Whether actin polymerisation is mechanistically related to the later activation of small GTPase has to be addressed in further studies. Evidence from cardiomyocytes indicates that the actin changes produced by cell stretching can secondarily activate RhoA and Rac1 small GTPases.^17^ Thus, it is possible that the small GTPase effects of PLY occur secondary to its actin rearrangement effects.

The interaction between actin and PLY is relatively strong (*K*_d_ of 170–190 nM) and is comparable to the binding affinity of phalloidin to F-actin (116 nM^18^) and of the Arp2/3 complex to F-actin (ATP bound, 40 nM; ADP bound, 1 μM^19^). After binding the membrane, the molecules of PLY distribute linearly in the monolayer of the membrane, as the linear concentration can reach very high values. The actin concentration directly beneath the membrane is also very high.^20^ Thus, the interface between the membrane and cytosol is molecularly favourable for a direct interaction between actin and PLY.

Consistent with the current knowledge about pore formation, a critical role in actin binding was observed for the membrane-penetrating portion of domain 3. The domain-3-containing fragment was the strongest actin-binding partner in the ELISA assays, and the deletion of alanine 146 and arginine 147 (the Δ6 mutation present in domain 3 blocks the toxin transition from a pre-pore to a pore and, therefore, the penetration through the membrane) in the full-length PLY molecule decreased its ability to bind and cluster actin at the GUV membrane. The decreased actin binding to the Δ6 mutant in the ELISA assay where no membrane was present, however, suggested that alanine 146/arginine 147 are also important for actin binding, irrespective of the presence of a membrane. Considering that these amino acids remain relatively high above the level of the membrane during pore formation,^2^ it is likely that they facilitate the refolding of domain 3 rather than directly interacting with actin. The membrane-penetrating β-strand hairpins (of domain 3) that build the β-barrel of the pore are most likely the effective actin-binding interfaces.

PLY is a critical pathogenic factor, aggravating the course of pneumococcal infections.^21,22^ It is difficult to judge the contribution to the progress of *S. pneumoniae* infection of either the actin-binding and stabilisation effects of PLY or its small GTPase activation in an isolated manner because these are always present with the formation of the pore by the toxin. However, even short-term modulation of actin turnover by PLY might affect the properties of actin-dependent structures of the organism (such as the blood–brain barrier and liver) and thus facilitate the propagation of *S. pneumoniae*. Indeed, knocking out PLY or neutralising the toxin by antibodies diminishes the invasive potential of pneumococci in model animals, prevents bacteraemia and inhibits the colonisation of various organs.^23,24^

In recent work,^25^ Vadia *et* *al.* describe the increased internalisation of beads and bacteria coated with the CDCs listeriolysin or PLY. The transmembrane actin binding by PLY that we observed could therefore contribute to the alterations in cell trafficking and the toxin-induced internalisation of particles. The internalisation of pneumococci is known to be important for the pathogenesis of the disease (e.g., for penetration through the blood–brain barrier).^26^ It is also possible that the interaction between PLY and actin initiates the endocytotic internalisation of the toxin, which interrupts actin binding and thus determines its transient nature. In cells, the interactions between actin and the actin-associated proteins are complex. PLY may also bind some of these complexes together with actin. Upon pore formation and subsequent calcium influx,^27^ the cleavage of some of these interaction partners (e.g., talin and/or vinculin) in a calcium-dependent manner (e.g., by calpain)^28^ could be another reason for the interruption of the PLY/actin interaction.

In conclusion, we demonstrate that PLY has the capacity to penetrate membranes in both cells and model bilayers in a manner that enables the toxin to interact with sub-membranous actin with a high affinity, which induces actin polymerisation. This interaction involves motifs in domains 1–3 of the toxin, which are also required for the pore-forming ability of PLY.^5^ Thus, PLY emerges as the first example of a pore-forming bacterial toxin that interacts directly with actin, which, together with its capacity to activate small GTPases, completes its profile as a specific actin cytoskeleton-remodelling factor.

## Materials and Methods

### PLY preparation

**Supplementary Fig. S3:**
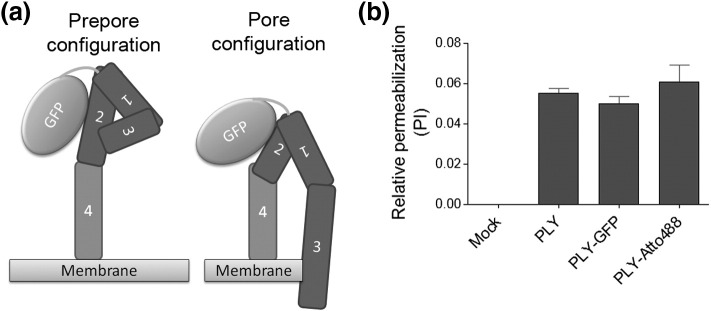
(a) Diagram of the position of the GFP tag at the N-terminus of PLY. (b) The lytic capacity of 0.05 μg/ml Atto488-labelled PLY and GFP-tagged PLY is equivalent to 0.1 μg/ml wild-type non-fluorescently labelled PLY in a propidium iodide permeabilisation live cell assay. This result is indicative of slightly improved pore formation by PLY in the presence of GFP and Atto488, most likely due to an improved monomer orientation.

Wild-type PLY and N-terminally GFP-tagged PLY (PLY-GFP) were expressed in *Escherichia coli* BL-21 cells (Stratagene, Cambridge, UK) and purified by metal affinity chromatography as described previously.^29^ The purified PLY was evaluated for the presence of contaminating Gram-negative lipopolysaccharide using the colorimetric Limulus amebocyte lysate assay (KQCL-BioWhittaker, Lonza, Basel, Switzerland). All purified proteins showed < 0.6 endotoxin units per microgram of protein. The initial stock of purified wild-type toxin exhibited an activity of approximately 5 × 10^4^ haemolytic units per milligram. The non-toxic Δ6 form of the toxin was constructed by site-directed mutagenesis (QuikChange SDM Kit, Stratagene), which deleted the amino acids alanine 146 and arginine 147 with the following deletion primers: 5′-GGTCAATAATGTCCCAATGCAGTATGAAAAAATAACGGCTC-3′ and 5′-GAGCCGTTATTTTTTCATACTGCATTGGGACATTATTGACC-3′. The properties of this mutant were examined in detail previously.^4,5^ The truncated mutants D123 (PLY containing domains 1, 2 and 3) and D4 (PLY containing domain 4) were purified in the same way. A detailed analysis of the lytic capacity of the GFP-tagged toxin confirmed that it behaved similarly to wild-type PLY (Supplementary Fig. S3).

### Cell culture

Primary astrocytes were prepared from the cortices of newborn C57BL/6 mice (postnatal day 3) as mixed cultures with microglia in Dulbecco's modified Eagle's medium (high glutamate) (Gibco, Invitrogen GmbH, Karlsruhe, Germany) supplemented with 10% foetal calf serum (PAN Biotech GmbH, Aidenbach, Germany) and 1% penicillin/streptomycin (Gibco). Fourteen days after being seeded in 75-cm^2^ cell culture flasks (Sarstedt AG & Co. KG, Nuembrecht, Germany), the cells were reseeded onto chamber slides (BD Falcon, Franklin Lakes, NJ, USA), chambered cover-glass bottom slides (Nunc/Thermo Scientific, Rockford, IL, USA) or dishes (Sarstedt) coated with poly-l-ornithine (Sigma-Aldrich Corp., St. Louis, MO, USA).

### Labelling of PLY with Atto488

Atto488, in the form of an NHS ester (Atto-Tec GmbH, Siegen, Germany), was added to PLY in a 4-fold molar excess [in 0.1 M *N*,*N*-bis(2-hydroxyethyl)glycine buffer, pH 9]. Alkaline conditions were maintained during the labelling reaction, which was performed for 30 min at room temperature (RT) in the dark. The excess dye was removed using a gel-filtration column (10 DG, BioRad, Germany). Covalent bonding was verified *via* SDS-PAGE analysis of the eluted fractions on a UV transilluminator (FluoChemQ, Alpha Innotech, Santa Clara, CA, USA). A detailed analysis of the lytic capacity of the labelled toxin confirmed that it behaved similarly to wild-type PLY (Supplementary Fig. S2).

### Immunofluorescence staining

Cells were fixed with 4% paraformaldehyde (Carl Roth, Karlsruhe, Germany) in phosphate-buffered saline (PBS) (pH 7.3) for 30 min, permeabilised with 0.1% Triton X-100 (Carl Roth) and blocked with 4% bovine serum albumin (Carl Roth). Actin staining was performed using phalloidin-Alexa555 (1:200) (Invitrogen).

### FRET detection in fixed cells *via* acceptor photobleaching

Primary mouse astrocytes were challenged with a sub-lytic concentration of PLY-Atto488, fixed and then stained with phalloidin-Alexa555 after permeabilisation on ice with 0.1% Triton X-100 for 3 min. The analysis was performed on a Leica LSM SP5 confocal microscope (Leica Microsystems, Mannheim, Germany) with 488 nm excitation of Atto488 and 561 nm excitation of Alexa555. The bleaching was performed with maximum laser power at 561 nm, and the images were analysed with the ImageJ software package (version 1.47a; National Institutes of Health, Bethesda, MD, USA).

### Preparation of G-actin and F-actin

To obtain G-actin, we reconstituted non-muscle actin (Cytoskeleton, Denver, USA) isolated from human platelets (> 99% pure) in 5 mM Tris–HCl, pH 8.0, 0.2 mM CaCl_2_, 0.2 mM ATP, 5% (w/v) sucrose, and 1% (w/v) buffer (Cytoskeleton). Monomeric actin was polymerised in 1:10 diluted APB (500 mM KCl, 20 mM MgCl_2_, and 10 mM ATP) (Cytoskeleton), followed by incubation of the solution at 24 °C for 1 h. Preformed F-actin was reconstituted in general actin buffer (5 mM Tris–HCl, pH 8.0, and 0.2 mM CaCl_2_) (Cytoskeleton).

### FRAP experiments

Non-muscle G-actin purified from human platelets containing covalently linked rhodamine at random surface lysine residues (Cytoskeleton) was used for the imaging of actin polymerisation. Actin-rhodamine was reconstituted in general actin buffer (see above) to a final concentration of 250 μg/ml. To achieve the polymerisation of actin, we added APB (Cytoskeleton). PLY was also used at a concentration of 250 μg/ml (actin/PLY ratio of 1:0.8) and allowed to polymerise at 37 °C for 1 h. Subsequently, 5 μl of the suspension was applied to a glass slide with a coverslip. Imaging was performed on a Leica LSM SP5 confocal microscope (Leica Microsystems). Bleaching was performed at 561 nm with maximum laser power and followed by scanning at 561 nm with a laser power of 10%. The analysis was performed with the ImageJ software package.

### Actin-binding spin-down assays

Freshly prepared F-actin (see above) was incubated with PLY or its mutant variants and fragments. The actin (4.6 μM) was incubated with the PLY variants (2.5 μM) for 1 h at 24 °C in a total volume of 100 μl, which was followed by centrifugation at 150,000***g***. The pellet containing actin was resuspended in 50 μl of MilliQ water. SDS sample buffer (containing β-mercaptoethanol, see above) was added to both fractions, and the samples were boiled and subsequently analysed by SDS-PAGE.

### ELISAs for the detection of protein interactions

F96 Cert. Maxisorp Immuno plates (Nunc/Thermo Scientific, Waltham, MA, USA) were coated overnight with PLY at concentrations between 5 and 300 μg/ml (in the stoichiometric ELISA experiments) or with 20 μg/ml of the PLY variants (in the domain interaction identification tests). The amount of adsorbed protein was evaluated and compared among different PLY forms/fragments by the copper iodide staining protocol for microtiter plates,^30^ and the actin absorption results were normalised to the adsorbed protein amount, as the coating differences never exceeded 20%. Nonspecific binding sites were blocked with 10% foetal calf serum (PAN Biotech GmbH) for 1 h. G-actin was added at a concentration of 20 μg/ml. After a 2-h incubation at RT, the wells were washed three times with 0.05% 1 × PBS-T (for PBS components, see above; Tween-20, Carl Roth). Then, an anti-actin antibody (1:1000; Cytoskeleton) was added, and the samples were incubated for 1 h at RT. Finally, a horseradish-peroxidase-conjugated goat anti-rabbit antibody (Jackson) was applied for 30 min, and the reaction was visualised using TMB (Nalgene/Thermo Scientific, Waltham, MA, USA).

### GUV experiments

GUVs were produced as described previously.^31^ Briefly, a mixture of 25% 1,2-Dioleoyl-sn-glycero-3-phosphocholine, 35% 1,2-Dipalmitoyl-sn-glycero-3-phosphocholine and 40% cholesterol (all from Sigma) in chloroform was employed for the electroformation of GUVs with a Vesicle Prep Pro device (Nanion Technologies GmbH, Munich, Germany) according to the manufacturer's instructions. GUVs were loaded internally with GFP-tagged PLY variants *via* cold fusion between the GUVs and protein liposomes at 4 °C.^16^ The efficiency of the fusion was verified using protein liposomes containing fluorescent secondary antibodies. The protein liposomes were prepared with the film hydration method using 40% DOPC and 60% DPPC, as described previously.^32^ Free, non-encapsulated PLY was captured *via* incubation for 2 h on a shaker platform with a cholesterol-containing trap that consisted of a dried multilamellar film composed of a cholesterol-containing lipid mixture, such as the mixture used to form the GUVs. The liposome mixture was tested for cytotoxicity and toxin binding, and it demonstrated a complete lack of free PLY. The fused GUVs demonstrated proper PLY-GFP staining localised on their surface. To verify the orientation of PLY-GFP on the surface of the GUVs, we performed a trypan blue assay (see Supplementary Fig. S3). For the actin polymerisation experiments, GUVs with or without PLY were incubated with rhodamine-labelled G-actin in a final mixture containing 7.5 μM G-actin, 160 nM Arp2/3 (Cytoskeleton) complex and 1 mM ATP APB^32^ (see Fig. 4a for a diagram).

### Statistical analysis

Statistical analysis was performed using GraphPad Prism 4.02 for Windows (GraphPad Software Inc., La Jolla, CA, USA). The statistical tests included a Mann–Whitney *U* test (comparing two groups differing in one parameter) and a one-way ANOVA with a Bonferroni post test (comparing three or more groups differing in one parameter).

The following are the supplementary data related to this article.

## Conflicts of Interest

The authors declare that they have no financial or other conflicts of interest.
